# The Relationship between Running Velocity and the Energy Cost of Turning during Running

**DOI:** 10.1371/journal.pone.0081850

**Published:** 2014-01-31

**Authors:** Yoichi Hatamoto, Yosuke Yamada, Hiroyuki Sagayama, Yasuki Higaki, Akira Kiyonaga, Hiroaki Tanaka

**Affiliations:** 1 Graduate School of Sports and Health Science, Fukuoka University, Fukuoka, Japan; 2 The Fukuoka University Institute for Physical Activity, Fukuoka, Japan; 3 Laboratory of Applied Health Science, Kyoto Prefectural University of Medicine, Kyoto, Japan; 4 Research Fellow (SPD), Japan Society for the Promotion of Science, Tokyo, Japan; 5 Faculty of Sports and Health Science, Fukuoka University, Fukuoka, Japan; West Virginia University School of Medicine, United States of America

## Abstract

Ball game players frequently perform changes of direction (CODs) while running; however, there has been little research on the physiological impact of CODs. In particular, the effect of running velocity on the physiological and energy demands of CODs while running has not been clearly determined. The purpose of this study was to examine the relationship between running velocity and the energy cost of a 180°COD and to quantify the energy cost of a 180°COD. Nine male university students (aged 18–22 years) participated in the study. Five shuttle trials were performed in which the subjects were required to run at different velocities (3, 4, 5, 6, 7, and 8 km/h). Each trial consisted of four stages with different turn frequencies (13, 18, 24 and 30 per minute), and each stage lasted 3 minutes. Oxygen consumption was measured during the trial. The energy cost of a COD significantly increased with running velocity (except between 7 and 8 km/h, p = 0.110). The relationship between running velocity and the energy cost of a 180°COD is best represented by a quadratic function (y = −0.012+0.066x +0.008x^2^, [r = 0.994, p = 0.001]), but is also well represented by a linear (y = −0.228+0.152x, [r = 0.991, p<0.001]). These data suggest that even low running velocities have relatively high physiological demands if the COD frequency increases, and that running velocities affect the physiological demands of CODs. These results also showed that the energy expenditure of COD can be evaluated using only two data points. These results may be useful for estimating the energy expenditure of players during a match and designing shuttle exercise training programs.

## Introduction

Ball sports such as soccer, basketball, handball, rugby, lacrosse and tennis place large metabolic demands on players. For example, video analysis of Italian “Serie A” matches showed that the average distance covered during 56 soccer matches was 10,950±1,044 m (range; 8,683 to 13,533 m) per player per match [1]. It is estimated that the total energy expenditure of a soccer player during one match is about 1,200–1,500 kcal [2]–[6], and these values include not only the energy utilized for the distance run, but also the energy requirements of other movements associated with soccer activities [7]. Professional soccer players in the FA Premier League perform more than 700 turns during a match [8]. Turning is a maneuver that includes a decrease and then an increase in velocity to change the velocity [9]. A COD while running requires applying additional force to the ground to direct the original momentum of straight running toward a new direction [10], [11]. Thus, CODs while running should require some additional energy.

There has been little research on the physiological response to a COD while running. A few previous studies have compared the physiological response to straight-line running with the response to shuttle running [12]–[15]. These studies showed that the inclusion of COD during submaximal [14] and high-intensity [13] running created a greater physiological demand (higher oxygen uptake [VO_2_]), heart rate [HR] and blood lactate [La]) than forward running without CODs. Although the results of these previous studies suggest that running with 180°CODs is more physiologically demanding than straight running, it is not clear what the actual energy cost of a COD is. Since CODs during running typically happen very quickly, it is difficult to estimate the energetic cost related to this maneuver.

We recently developed the different frequency accumulation method (DFAM) for evaluating the physiological demands of turning while running. This method is a graded test in which subjects perform 180° turns at different frequencies while running at a fixed average velocity; this allows estimation of the energy cost of turning by measuring the oxygen consumption and comparing it with that of steady-state [16]. However, we initially investigated the energy cost of turning while running at two low velocities (4.3 and 5.4 km/h). Thus, further study is needed to examine the relationship between running velocity and the energy cost of a COD (i.e., whether the energy cost is affected by running velocity) and to quantify the energy cost of a COD at higher velocities.

The aims of this study were 1) to compare the physiological demands of straight-line running and running with 180°CODs, 2) to examine the validity of the DFAM to calculate the energy cost of a change of direction, and 3) to establish an equation describing the energy cost of a 180°COD as a function of running velocity.

## Methods

### Subjects

Nine male university students who were well-trained lacrosse players and practiced 5 days per week volunteered to participate in this study. Table 1 shows the descriptive characteristics of the subjects. Subjects had been practicing lacrosse for more than 8 months, but before starting lacrosse they had played ball games such as volleyball, baseball, tennis, and basketball) for more than 6 years; therefore, it was expected that this population would be familiar with performing CODs while running. All of them were free of any injury that might influence their athletic performance. The subjects were advised to abstain from strenuous exercise on the day before each experiment and to maintain their normal daily nutritional intake during the study. This study was approved by the Ethics Committee of Fukuoka University, Fukuoka, Japan (12-02-02), and written informed consent was obtained from all participants. This study followed the principles of the World Medical Association Declaration of Helsinki, the Ethical Principles for Medical Research Involving Human Subjects, and the Ethical Guidelines for Epidemiological Research provided by the Ministry of Education, Culture, Sports, Science and Technology and Ministry of Health, Labour and Welfare, Japan.

**Table 1:** Characteristics of experimental subjects (n = 9).

Age (years)	Height (cm)	Weight (kg)	VO_2_ peak (ml/kg/min)
20.6±1.2	169.6±3.6	65.9±9.3	58.0±5.5

### DFAM

Our previous study established the DFAM as a novel approach to evaluating the instantaneous physiological demands of turning while running. Using this method, we found that the gross energy expenditure (EE) increased linearly with COD frequency (Figure S1). The EE of a COD (turn cost) was expressed as the slope of the regression of gross EE versus turn frequency, and the intercept of the regression line was the EE of running at a constant velocity. The EE of a turn included the linear deceleration to slow down the forward velocity as the runner initiated the COD, and the linear acceleration to get the runner back up to the target running velocity after the body had been rotated. Thus, this method made it possible for us to calculate the net EE of a turn while at a constant running velocity.

### Experimental Protocols for Assessing the EE of a COD

Each subject performed six shuttle exercise trials at different average running velocities. The running velocities in this study were 3, 4, 5, 6, 7 and 8 km/h. The CODs were performed using the sidestep cutting technique, in which the runner turns away from the side of the supporting leg [10], [11], [17]. The first trial was conducted after more than 2 days of instruction and practice, and before every trial the subjects were reminded how to perform the 180°CODs and practiced the turning technique for a few minutes. The trials were conducted over a one-month period and the order in which the participants performed the trials was randomly determined. If 3- or 4-km/h shuttle exercise was selected, then another trial was performed after taking a rest of at least 20 min. All other trials were performed on separate days. All participants were instructed to get at least 6 hours’ sleep before the test days and to avoid food, caffeine, tobacco products, and alcohol for 3 hour prior to the trials, and were asked to wear the same indoor sports shoes each time. The experiments were conducted in an indoor facility with polyvinyl chloride flooring, and the temperature during the experiments ranged between 22 and 24°C.

**Figure 1:**
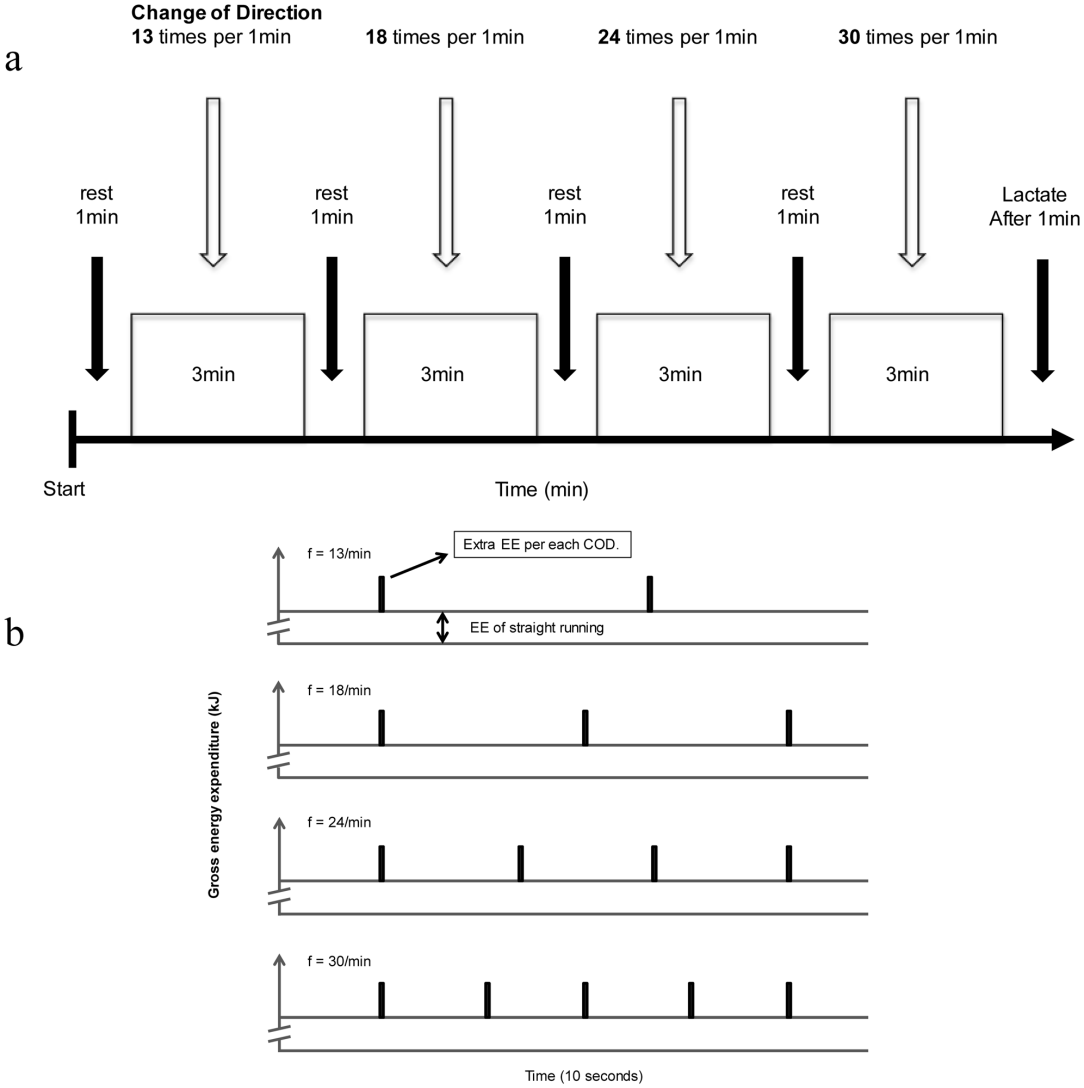
(a) Shuttle exercise protocol. Each stage lasted 3 min, with a 1-min rest between stages. COD frequencies of each stage were 13, 18, 24 and 30 CODs per minute. (b) EE of a COD while running. Extra energy expenditure occurs every time a COD is performed. The figure shows the estimation for all turn frequencies over 10 seconds. COD, change of direction; EE, energy expenditure.

The trial protocol is shown in Figure 1. Each trial consisted of four stages of different 180°COD frequencies. Each stage lasted three minutes, with a one-minute rest between stages. The COD frequencies in each stage were 13, 18, 24 and 30 per minute.

However, the 8 km/h only trial had three stages and the COD frequencies were 13, 18, and 30 per minute. CODs were performed so that the runner turned an equal number of times in both directions. Running distances were determined by the turn frequencies and average running velocities. A metronome was used to pace the participants at average running velocities and to indicate the moment of COD (DM-17; Seiko Digital Metronome, Seiko Corp, Tokyo, Japan). Participants were asked to run a certain distance just within determined beats, but at their own preferred stride length, which we particularly did not determine, and they ran back and forth freely. For example, when subjects performed the 180°CODs 30 times at each speed, they had to run the distances determined by each running speed in just 2 seconds, and perform CODs at the same time (i.e: when the subject performed the 180°CODs 30 times at 6 km/h (100 m/min), the running distance was 3.33 m with CODs every 2 seconds (60 beats per minute : bpm, 1 beat is 1 second). Thus, the metronome was used to provide an auditory key to the subjects to fix the timing of CODs and to regulate the running pace exactly and they were allowed to use their own preferred stride length and frequency.

Gas exchange measurements were carried out during the trials (ARCO 2000, ARCO System, Chiba, Japan). VO_2_ was assessed for the final 1 minute of each stage of the trial. Heart rate (HR) was measured during the last 30 seconds with a Polar heart rate monitor (CE0537, Polar Electro, Kempele, Finland). Subjects were asked to provide a rating of perceived exertion (RPE) using the Borg scale [18] after each stage. A blood sample was collected from the earlobe for determination of blood lactate concentration (La) at one minute after each trial. These samples were collected after cleaning the earlobe with alcohol and were immediately analyzed using a portable blood lactate analyzer (Lactate pro, Arkray, Japan).

### Aerobic Capacity Test

The aerobic capacity test consisted of 6 incremental velocity stages (from 3 to 8 km/h) and ramp increments during treadmill exercise. This test had two purposes: 1) to determine steady-state oxygen consumption at from 3 to 8 km/h and 2) to determine peak oxygen uptake (VO_2_ peak). The aerobic capacity test was conducted within one month after the EE test. Subjects started with a warm-up phase consisting of 2 min of walking at 3 km/h. After the warm-up, treadmill velocity was then increased by 1 km/h every 3 min until 8 km/h was reached (6 stages total). Thereafter, to measure VO_2_ peak, running velocity was immediately increased to 10 km/hr, then increased by 1 km/hr every 1 min until reaching12 km/h. Following this, the velocity of 12 km/h was held constant while the treadmill grade was increased by 2% every 1 min. The test was continued until subjective exhaustion was achieved, and VO_2_ values were recorded continuously throughout the trial. Expired gas was analyzed by mass spectrometry (ARCO 2000, ARCO System, Chiba, Japan). The highest VO_2_ over 1 minute was regarded as the VO_2_ peak. We also obtained the HR value during the last 30 seconds and RPE immediately after the test and the La value after 1 min. VO_2max_ was assumed to be reached when the oxygen uptake plateaued or two of the following four criteria were achieved: 1) reaching at least 8 mmol/L La concentration; 2) reaching the age-adjusted 90% of maximal HR; 3) reaching at least an RPE value of 18; or 4) reaching a respiratory exchange ratio (RER) greater than 1.10 [19]. The subjects in the study fulfilled two of four criteria (La: 9.0±2.2, RPE: 19.1±0.6, HR 194.4±8.4, RER: 1.13±0.06).

### Energetic Measurements

EE during both exercise tests was measured by collecting an expired gas sample through a facemask. Respiratory gas analysis was conducted using the mixing chamber method to evaluate the volume of expired air, and the O_2_ and CO_2_ fractions were analyzed by mass spectrometry (ARCO 2000, ARCO System, Chiba, Japan) every 12 seconds and averaged to 1 min. At the beginning of each trial the system was calibrated using a 3-L calibration syringe for volume calibration, and two different gas mixtures of known concentrations (20.93% O_2_ and 0.04% CO_2_; 15.00% O_2_ and 4.55% CO_2_) for calibration of the gas analyzers.

### Statistical Analyses

All analyses were conducted using SPSS software version 20 (SPSS, IBM, Armonk, NY, USA). All values are expressed as mean±standard deviation (SD). Linear regression analyses were used to calculate slopes and intercepts for gross EE against turn frequency at each running velocity. One-way ANOVA was used to compare the slope of the VO_2_ that indicates the cost of a COD performed during shuttle exercise at different running velocities. Post-hoc Bonferroni tests were used to determine the significance of differences. Regression line and curve analysis was performed to predict the cost of CODs at different running velocities and expresses the relationship between the energy cost of turning and running velocity. These regression equations were based on the average data for all subjects at running velocities of 3–8 km/h. The six data points for running velocity were plotted on the x-axis and the energy cost of COD values were plotted on the y-axis. The relationship between the VO_2_ of treadmill running and VO_2_ of the intercept of regressions between VO_2_ and COD frequency at 3–8 km/h were determined by Pearson’s product-moment correlation coefficients and compared by paired t-test. Differences were considered significant at an alpha level of P<0.05.

## Results

**Figure 2:**
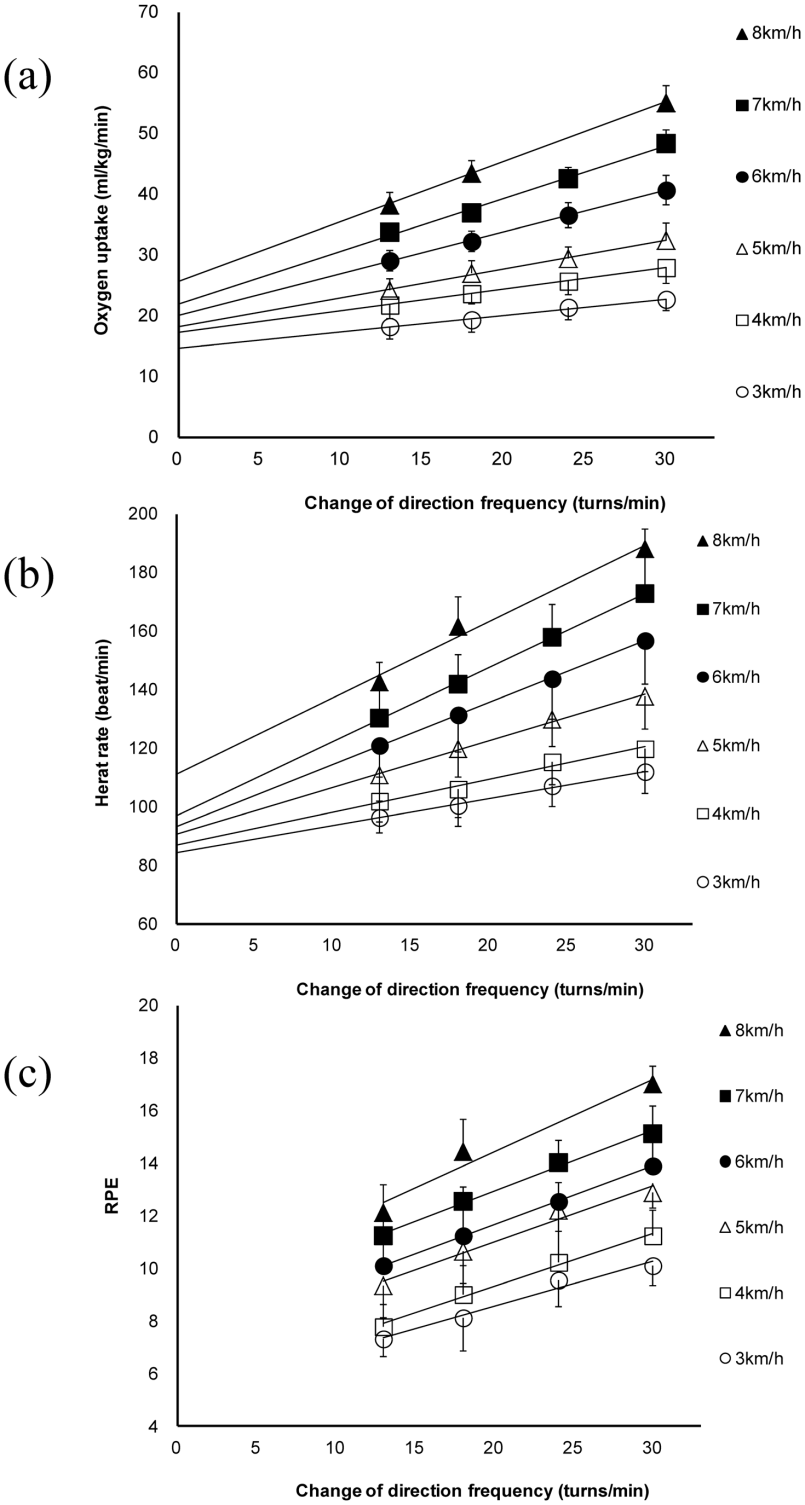
Comparison of physiological responses and RPE while running at different velocities. Relationship between turn frequency and oxygen consumption (A), heart rate (B), and RPE (C), while running at different velocities. HR, heart rate; RPE, rating of perceived exertion; VO_2,_ gross oxygen consumption.

**Figure 3:**
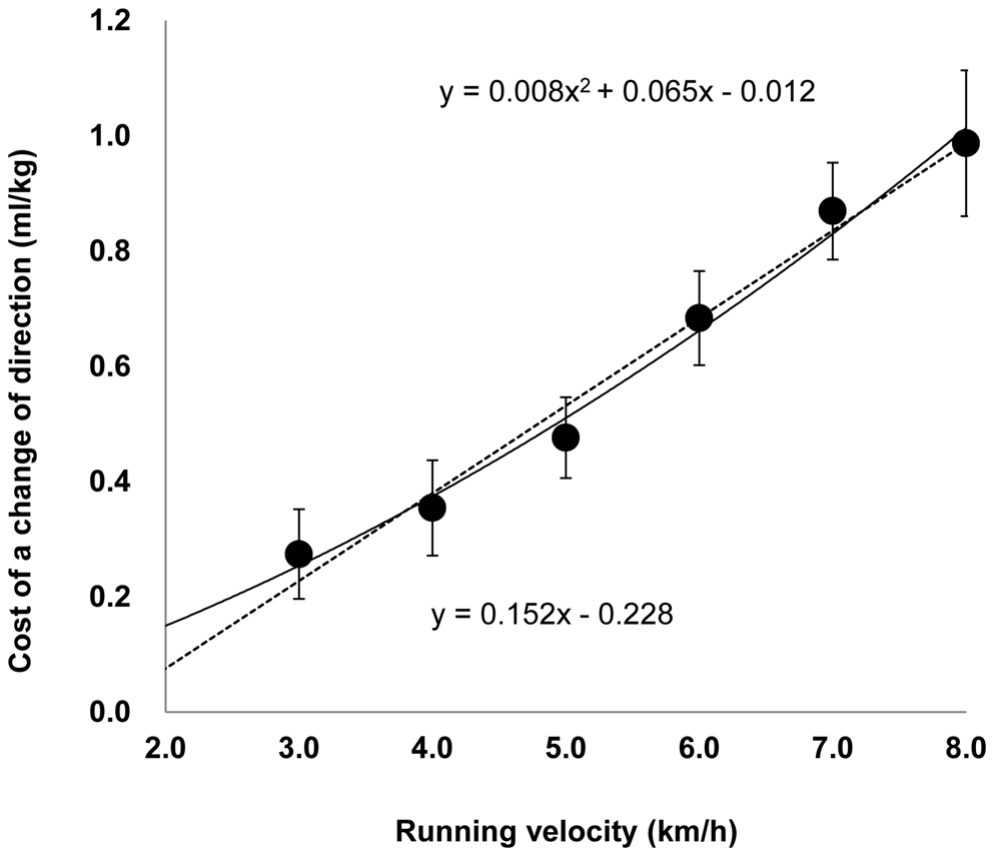
Relationship between running velocities and energy cost of a turn. Values are averages. The relationship was expressed by both an approximate quadratic (r = 0.994, p = 0.001, solid line) and a linear model (r = 0.991, p<0.001, dashed line).

All participants successfully completed all trials. Table 2 shows the gross VO_2_ of different COD frequencies and the VO_2_ of treadmill running at 3–8 km/h running velocities. The linear regressions of COD frequency and VO_2_, HR, and RPE were obtained for each running velocity (Figure 2). As running velocity increased, the energy cost of a COD also increased. At running velocities of 3, 4, 5, 6, 7, and 8 km/h, the energy cost of a COD was 0.27±0.03, 0.35±0.05, 0.48±0.10, 0.68±0.08, 0.87±0.13, 0.99±0.14 (ml/kg), respectively (Figure 3). The cost of changing direction did not differ between running velocities of 7 and 8 km/h (7 vs 8 km/h, p = 0.110).

**Table 2:** Mean gross VO_2_ at different COD frequencies and the VO_2_ of treadmill running at velocities of 3 to 8 km/h.

Running velocitieskm/h	Treadmill VO_2_(ml/kg/min)	13 turns/min(ml/kg/min)	18 turns/min(ml/kg/min)	24 turns/min(ml/kg/min)	30 turns/min(ml/kg/min)
3	13.4±1.0	18.2±1.9	19.3±2.0	21.4±2.0	22.7±1.8
4	15.2±1.3	21.9±2.4	23.8±1.8	25.8±2.4	28.0±2.5
5	18.7±1.3	24.2±1.9	27.1±2.1	29.4±2.0	32.5±2.9
6	22.0±1.2	29.2±1.7	32.3±1.7	36.6±2.1	40.8±2.5
7	24.8±1.2	33.9±1.4	36.9±1.1	42.6±1.9	48.4±2.3
8	27.3±1.5	38.4±1.9	43.6±2.1	–	55.2±2.7

COD, change of direction; VO**_2_**, oxygen consumption.

The average energy cost of a COD versus running velocity in all subjects was best expressed by a quadratic model (y = −0.012+0.066x +0.008x^2^, [r = 0.994, p = 0.001]), but was also well expressed by a linear model (y = −0.228+0.152x, [r = 0.991, p<0.001]). Blood lactate values 1 min after each trial were 1.0±0.2, 1.1±0.2, 1.2±0.4, 1.7±0.6, 2.8±0.8, 4.7±1.7 ml/kg at 3–8 km/h, respectively.

**Figure 4:**
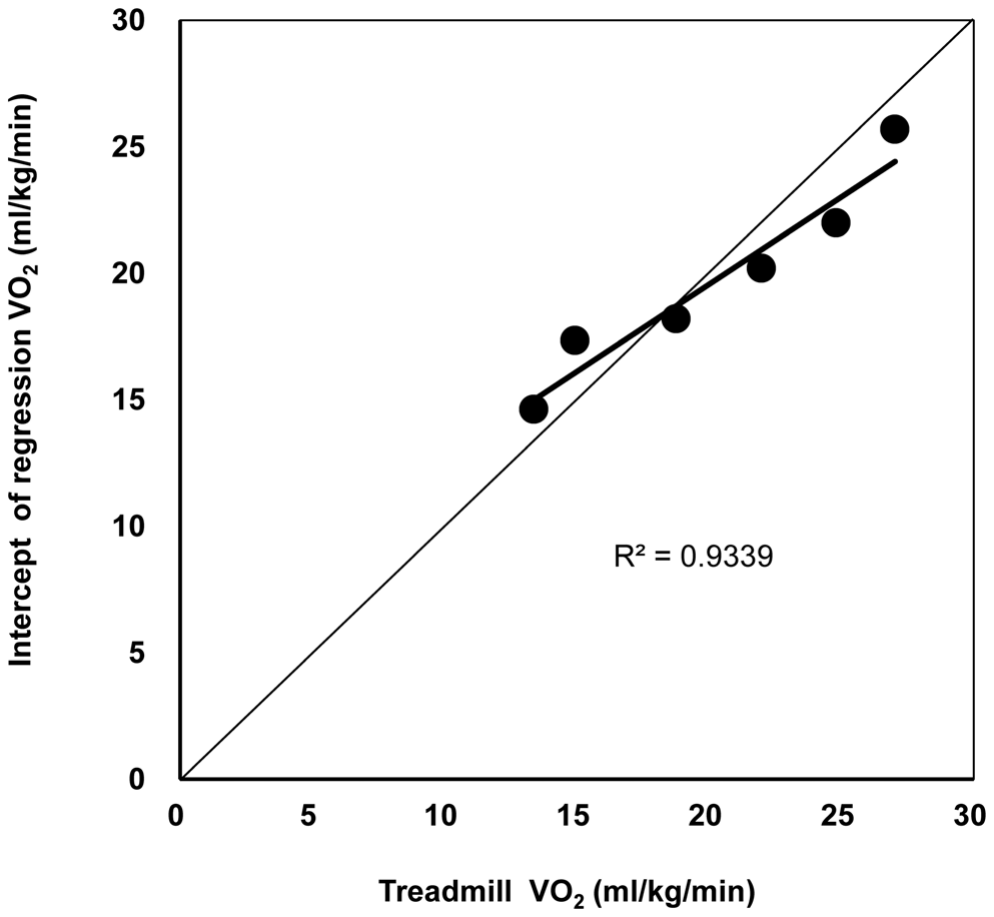
Relationship between actual and estimated running VO_2_ at different running velocities. The VO_2_ of straight running and the intercept of the linear regression VO_2_ at 6 different running velocities (3–8 km/h) were significantly correlated (r = 0.966, p = 0.002). VO**_2_**, gross oxygen consumption.

In order to confirm the validity of the hypothesis that the intercept of the regression line of VO_2_ versus COD frequency corresponds to the VO_2_ of treadmill running, we examined the correlation and conducted paired t-tests to assess the relationship between the VO_2_ of treadmill running and the intercepts of the regression line for each running velocity. There was a significant correlation between the VO_2_ of treadmill running and the intercepts of the regression of VO_2_ versus COD frequency at each running velocity (r = 0.966, p = 0.002) (Figure 4), and these were not significantly different (paired t-test, p = 0.582).

## Discussion

The purpose of this study was to investigate the difference in energy demand between running with 180°CODs and straight-line running and, in particular, the influence of running velocity on the VO_2_ associated with a 180°COD and the validity of the DFAM. We also suggest a protocol for measuring the energy cost of a turn more easily. The results of this study show that as running velocities increase, physiological responses such as HR, RPE, La, and the VO_2_ of CODs also increase.

In recent years a few studies have focused on different physiological responses to running with 180°CODs and running without turning. Dellal et al. (2010) compared physiological responses such as HR and La in soccer players during intermittent straight-line running and intermittent shuttle exercise with 180° turns performed at vVO_2max_ (maximal aerobic velocity) running velocities and covering the same distances [13]. The values of HR and La during shuttle exercise were higher than those during straight running. When running velocities were adjusted for maximal O_2_ uptake during a straight-line incremental protocol, the pulmonary VO_2_ for shuttle running was higher than for straight running [14]. A recent study also showed that when comparing shuttle exercise over a 3.5-m and a 7.0-m course at the same average running velocities and for the same total distances covered, the 3.5-m shuttle exercise induces a greater physiological response [20]. This occurs because of the greater number of 180°CODs required for the 3.5-m course. Our results also indicate that the VO_2_ responses to running with turning were greater than for straight running at each running velocity (Table 2). Table 3 illustrates the values of VO_2_ at four different COD frequencies for the same average running velocities and compares them to treadmill running at the same velocites. The gross VO_2_ of running including 30 CODs/minute was approximately twice the gross VO_2_ of treadmill running at the same velocity. For example, in general, 3 km/h is a very low running velocity, but 30 CODs per minute at 3 km/h has similar metabolic demands to straight running at 6 km/h. In addition, the VO_2_ at 8 km/h with 30 CODs per minute was close to VO_2max_, although a running velocity of 8 km/h would be classified as “low-intensity” activity in a ball game [21]. The estimated energy cost during the acceleration phase of running is higher than the energy cost while running at a constant velocity [22]. A COD while running requires a phase of deceleration and acceleration and eccentric and concentric muscle contraction [9], which generates a greater physiological load [12], [13], [14], [23]. These results indicate that running with CODs requires extra energy, even when running at a very low velocity.

**Table 3:** Comparison of straight and shuttle running velocites for the same VO_2_ demands.

Running	Treadmill	13 turns/min	18 turns/min	24 turns/min	30 turns/min
velocity km/h	(km/h)	(km/h)	(km/h)	(km/h)	(km/h)
3	3.1	4.8	5.2	5.9	6.4
4	3.8	6.1	6.7	7.4	8.2
5	5.0	6.9	7.8	8.7	9.7
6	6.1	8.6	9.7	11.1	12.5
7	7.1	10.2	11.2	13.2	15.2
8	7.9	11.7	13.5	–	17.5

VO**_2_**, oxygen consumption.

One of the findings of this study is the equation demonstrating the relationship between VO_2_ and COD frequency, which allows the energy cost of a 180°COD while running at different velocities to be quantified. Our results show a linear relationship between gross VO_2_ and COD frequency at running velocities of 3–8 km/h, and the slope of the regression line indicates the energy cost of a COD while running [16]. Also, the cost of a COD increased as running velocity increased. The estimation of the energy cost of a COD is expressed by the regression equations of the relationship between energy cost of a COD and running velocities (Figure 3). Although this relationship is best represented by a quadratic function (r = 0.994), it is not similar to that of the linear regression equation at 3–8 km/h (r = 0.991). The values that we reported for the energy cost of a COD at the two running velocities used in our previous study were comparable to the values obtained from the quadratic equation of the relationship between running velocity and turn cost at similar running velocities in this study. The mean VO_2_ of a turn in the previous study was 0.34±0.13 ml/kg at 4.3 km/h and 0.55±0.09 ml/kg at 5.4 km/h. When the same running velocities were used in the quadratic equation, the VO_2_ of a 180° turn was similar to these previously reported values, (0.42 ml/kg at 4.3 km/h and 0.57 ml/kg at 5.4 km/h). At the level of the individual participants, both linear and curved relationships between the EE of a COD and running velocity were observed. The energy cost of a COD may differ between individuals because of differences in COD technique, stature and training volume [14], [24]. It is possible that the energy cost of a COD performed by ball game players may be lower than that of players of other sports because ball game players perform CODs regularly, which may affect the results. Thus, the equation we have identified is most likely suitable for ball game players.

As running velocity increases, the blood lactate values increase. An earlier study has reported similar results [13]. The 180°COD with a higher running velocity would require more energy to change the velocity (for both deceleration and acceleration) and additional muscular action, possibly inducing a glycolytic contribution [13]. The standard deviations of blood lactate values also enlarge as running velocity increases. There will be less deviation among baseline La values and more deviation as La accumulates. This is probably because blood lactate accumulation is highly related to VO_2_ peak [25]. The VO_2_ peak of subjects in this study ranged from 47.0 to 63.8 ml/kg/min; therefore, blood lactate accumulation would differ between individuals the same exercise intensity. Further studies of the differences in energy cost of turning in players of various sports and body compositions are needed.

We hypothesized that the intercept of the regression line between gross VO_2_ and turn frequency would correspond to the VO_2_ of a steady-state at a constant running velocity. In our previous study we did not compare the VO_2_ of the intercept to the actual VO_2_ of forward running at steady state. In this study, the VO_2_ was measured at each running velocity on the treadmill, and the VO_2_ of the intercept was then compared with the measured value. It is well known that VO_2_ increases linearly as running velocity increases [26], [27]. Our results demonstrate that the VO_2_ of the regression intercept also increases with running velocity. There was a strong correlation between the VO_2_ of the intercepts and the measured VO_2_ (Figure 4), and these values did not significantly differ (paired t-test, p = 0.582). These data suggest that the intercept of the regression line very closely approximates the actual VO_2_ of steady state running at the same running velocity and confirm that the DFAM is a reasonable method for evaluating the energy cost of CODs while running.

**Figure 5:**
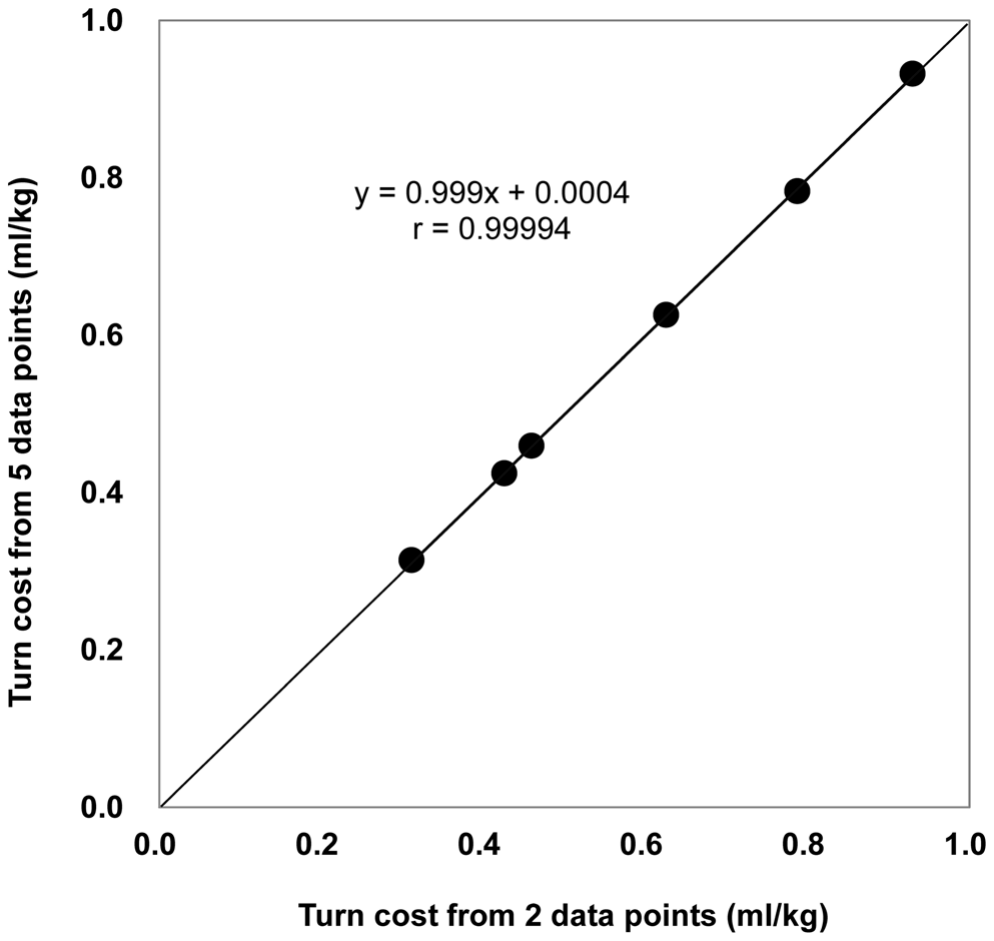
Comparison of turn cost for different data points. There was strong correlation (r = 0.99994, p<0.00001) between the slopes of regression lines drawn using two data points and five data points to evaluate the energy cost of a COD. The slope of the regression line of gross VO_2_ and the graded COD frequency test indicates the energy cost of a COD while running. COD, change of direction; VO**_2_**, gross oxygen consumption.

In addition, both the previous study and the present one show a linear relationship between gross VO_2_ and turn frequency. Therefore, turn cost can be calculated easily from only two data points (i.e., the cost of a COD can be evaluated by one treadmill and one turn trial session). We compared the energetic cost of a COD calculated using two data points (VO_2_ of treadmill running and 30 times COD frequency) with the cost of a COD calculated using five points (VO_2_ of treadmill run and running with four different turn frequencies) at 3–8 km/h (Figure 5); there was a significant correlation between both calculated values (r = 0.99994, p<0.00001) and they were not significantly different (p = 0.694). These results indicate that the energy cost of a turn can be calculated accurately using only two data points. This will facilitate further investigation of the energy costs of CODs at various running velocities.

One method for estimating the EE of soccer players is to calculate it from only the distance covered by the player during a single soccer match [28]. However, some researchers have commented that estimating EE from distance covered may underestimate the actual value because extra energy demands associated with soccer activities such as turning, jumping, dribbling and performing soccer skills are not accounted for [7], [29], [30]. This suggests the need to include the energy costs of ball handling and additional energy cost of movement. Reilly and Ball demonstrated the additional energy cost of dribbling a ball on a treadmill compared with running at the same speed alone [31]. In this study, we relate the additional energy cost of turning while running by fitting an equation to our measured EE from turning at different running velocities. This equation may allow for corrections to be made to the EE calculated from distance covered in soccer match and account for underestimation of the extra energy costs of maneuvers associated with the ball game.

In summary, we used expired gas samples to measure the physiological response to running with 180°CODs under steady-state conditions. Running with CODs was more physiologically demanding than straight running at the same average running velocities. These results also provide further confirmation that the DFAM is a reasonable method for investigating the energy cost of CODs, and that running velocity affects the energy cost of CODs. Our results also suggest that the energy cost of a COD can be calculated using only two VO_2_ measurements.

## Supporting Information

Figure S1 **Relationship between gross energy expenditure and COD frequency.** COD, change of direction; EE, energy expenditure; α, EE of a 180°COD at one running velocity; f, COD frequency; running EE: EE at constant velocity.
(TIF)
